# Update on resistance status of *Anopheles gambiae s.s. *to conventional insecticides at a previous WHOPES field site, "Yaokoffikro", 6 years after the political crisis in Côte d'Ivoire

**DOI:** 10.1186/1756-3305-5-68

**Published:** 2012-04-02

**Authors:** Alphonsine A Koffi, Ludovic P Ahoua Alou, Maurice A Adja, Moussa Koné, Fabrice Chandre, Raphael N'Guessan

**Affiliations:** 1 Institut Pierre Richet (IPR), BP 47 Abidjan, Côte d'Ivoire; 2 Laboratoire de Zoologie et Biologie Animale, Université de Cocody, 22 BP 582 Abidjan 22, Côte d'Ivoire; 3 Institut de Recherche pour le Développement (IRD)/Laboratoire de Lutte Contre les Insectes Nuisibles (LIN) LIN, IRD/UR 016, 911 Ave Agropolis, 34394 Montpellier Cedex 5, France; 4 London School of Hygiene and Tropical Medicine, Keppel Street, London WC1E 7HT, UK; 5 Centre de Recherche Entomologique de Cotonou, Cotonou 06 BP 2604, Bénin

## Abstract

**Background:**

At Yaokoffikro field site near Bouaké, in central Côte d'Ivoire, a group of experimental huts built in 1996 served over many years for the evaluation of insecticides against highly resistant mosquitoes. Breeding sites of mosquitoes and selection pressure in the area were maintained by local farming practices until a war broke out in September 2002. Six years after the crisis, we conducted bioassays and biochemical analysis to update the resistance status of *Anopheles gambiae s.s. *populations and detect other potential mechanisms of resistance that might have evolved.

**Methods:**

*An. gambiae s.s. *larvae from Yaokoffikro were collected in breeding sites and reared to adults. Resistance status of this population to insecticides was assessed using WHO bioassay test kits for adult mosquitoes with seven insecticides: two pyrethroids, a pseudo-pyrethroid, an organochloride, two carbamates and an organophosphate.

Molecular and biochemical assays were carried out to identify the L1014F *kdr *and *ace-1^R ^*alleles in individual mosquitoes and to detect potential increase in mixed function oxidases (MFO), non-specific esterases (NSE) and glutathione S-transferases (GST) activity.

**Results:**

High pyrethroids, DDT and carbamate resistance was confirmed in *An. gambiae s.s. *populations from Yaokoffikro. Mortality rates were less than 70% with pyrethroids and etofenprox, 12% with DDT, and less than 22% with the carbamates. Tolerance to fenitrothion was observed, with 95% mortality after 24 h.

PCR analysis of samples from the site showed high allelic frequency of the L1014F *kdr *(0.94) and the *ace-1^R ^*(0.50) as before the crisis. In addition, increased activity of NSE, GST and to a lesser extent MFO was found relative to the reference strain Kisumu. This was the first report detecting enhanced activity of these enzymes in *An. gambiae s.s *from Yaokoffikro, which could have serious implications in detoxification of insecticides. Their specific roles in resistance should be investigated using additional tools.

**Conclusion:**

The insecticide resistance profile at Yaokoffikro appears multifactorial. The site presents a unique opportunity to evaluate its impact on the protective efficacy of insecticidal products as well as new tools to manage these complex mechanisms. It calls for innovative research on the behaviour of the local vector, its biology and genetics that drive resistance.

## Background

The scaling up of Long Lasting Insecticidal Nets (LLINs) and to some extent Indoor Residual Spraying (IRS) is a major element of international strategies to control malaria, particularly in sub-Saharan Africa [1]. Pyrethroids are the only class of insecticide currently recommended for use on LLINs [2]. During the last decade pyrethroid resistance has become widespread in *Anopheles gambiae s.s. *in sub-Saharan Africa [3-5], probably as a consequence of use of pyrethroids in agriculture [6,7] but also increasingly through exposure to LLINs, as coverage is scaled up [8,9]. Even for IRS, with only four insecticide classes currently available and resistance reported to all four of these in some populations of *An. gambiae s.s. *[10], the options for managing resistance and providing sustainable vector control with existing chemicals are limited. Recent product development partnership has been established to stimulate the search for alternative active ingredients or improved formulations of insecticides for vector control, and several promising leads are being evaluated in laboratory and field trials [11]. Because of the hectic and burdensome regulation process to develop these products, it may take several years before many of these come onto the market. Meanwhile, Industry must continue to produce new prototype LLINs to ensure wider community coverage until new weapons are available.

Experimental huts constitute a label for the WHO Pesticide Evaluation Scheme (WHOPES) to grant phase II approval of insecticidal products after they have satisfied a number of entomological criteria in different settings [12]. Given the patchy distribution of pyrethroid resistance evolving in *Anopheles *vectors, the choice and location of a field site for product evaluation must fulfil specific characteristics, such as the resistance pattern of local vector population, their abundance and the genetic structure of such populations. Under the auspice of WHOPES, field sites to evaluate insecticides have been established in a few countries where varying resistance mechanisms to insecticides occur. This includes Cameroon [13], Vietnam [14], Burkina Faso [15], Côte d'Ivoire [16] and Benin [17].

In Côte d'Ivoire, an armed conflict broke out in September 2002, that caused a lot of population movement across the country. The resistance levels in mosquitoes at the field site, Yaokoffikro, are maintained by local farmers producing year round vegetables for local consumption. It is unclear whether such movement was accompanied by a desertion in farming practices that might have led to a shift in selection pressure. Six years after the crisis, we conducted bioassays and biochemical analysis to update the resistance status in *An. gambiae *and detect other potential mechanisms of resistance that might have evolved.

## Methods

### Study area

Yaokoffikro, a suburban village of Bouaké city in central Côte d'Ivoire, geo-referenced at 5°1' W longitude and 7°11' N latitude. The village is situated along a large valley producing rice and vegetables for local consumption. These farming practices constituted suitable breeding sites for mosquitoes. A group of experimental huts belonging to the "Institut Pierre Richet (IPR)" were constructed in 1998 at the site and served over many years for the evaluation of different insecticides under the auspices of WHOPES [18-24].

Mosquito population in the area is composed of *An. gambiae s.s.*, *Culex sp. *and *Mansonia sp. An. gambiae s.s. *is mostly S molecular form [25,26] and strongly resistant to pyrethroids and DDT with the Leu-Phe *kdr *mutation (L1014F *kdr*) showing allelic frequency above 0.90 [16,24,27,28]. Resistance to carbamates and organophosphates involving the *ace-1 *G119S mutation *(ace-1^R^) *was also highly expressed in this species with allelic frequency averaging 0.45 [24,29,30].

The style of the huts, typical of the region has been widely described in previous trials conducted at the site [31-33].

### Mosquito collection

During the rainy season in June 2008, larvae of *An. gambiae s.s. *were collected at the site and reared at IPR insectary for emergence and testing of adults. An insecticide susceptible *An. gambiae s.s. *Kisumu served as a reference strain.

### Insecticide susceptibility tests

Susceptibility bioassays on adult mosquitoes were conducted using WHO test kits [34]. Diagnostic concentrations of seven insecticides of technical grade quality belonging to different chemical classes were prepared and tested as follows:

- permethrin 25/75 (1%) (Agrevo, Berkhamsted, UK) and lambdacyhalothrin (0.05%) are pyrethroids obtained from Syngenta, UK;

- etofenprox (0.05%), a pseudo-pyrethroid from Mitsui Toatsu, Japan;

- DDT (4%), an organochlorine from Syngenta; carbosulfan (0.4%) from FMC, Philadelphia, USA and propoxur (0.1%) from Bayer,

- Leverkusen, Germany, are both carbamates;

- fenitrothion (1%), an organophosphate purchased at Sigma-Aldrich, St Louis, MO, USA.

Impregnated papers were prepared in our laboratory using technical grades of the above insecticides dissolved via acetone in silicone oil 556 (Dow Corning, Midland, MI, U.S.A) as a carrier. Treatment of the filter paper was made on the basis of 3.6 mg of oil per cm^2^. Whatman filter papers (12 cm × 15 cm) were impregnated with a mixture of 0.7 mL silicone oil + 1.3 mL insecticide acetonic solution. Papers were stored at 4°C and used no more than three times.

Tests were performed with batches of 25 unfed females of *An. gambiae s.s.*, 3-5 days old, four replicates per concentration. Mosquitoes were exposed to the insecticide treated papers for 60 min at 25 ± 2°C and 80% RH. After the exposure period, all the mosquitoes were transferred to the observation tube of the test kit, supplied with honey solution and held for 24 h before scoring mortality. Batches exposed to untreated papers were used as control.

Field samples were compared to a susceptible reference strain of *An. gambiae s.s. *Kisumu. All control survival specimens (including the susceptible reference mosquito) from the tests were frozen at -80°C for biochemical analysis. The exposed samples of mosquitoes to the different insecticides were kept in the fridge for molecular analysis.

### PCR detection of the L1014F *kdr *and *ace-1 *G119S mutations

Genomic DNA was extracted from individual *An. gambiae s.s.*, following the method of Collins *et al. *[35]. This was used for the detection of the L1014F *kdr *as per Martinez-Torres *et al. *[36] and the *ace-1 *G119S mutation (*ace-1^R^*) as per Weill *et al. *[37].

### Biochemical analysis

Biochemical assays were performed to compare the levels of activity of mixed function oxidases (MFO), non-specific esterases (NSE) using α-naphtyl acetate as a substrate and glutathione S-transferases (GST) [38] in the *An. gambiae s.s. *susceptible Kisumu and the field population from Yaokoffikro. Activity of AChE insensitivity was also measured and compared between Kisumu and Yaokoffikro mosquitoes following the method by Hemingway *et al. *[38]. Mosquitoes used for the biochemical analysis have not been exposed to any insecticides prior to the assay.

### Data analysis

WHO [34] criteria was adopted for distinguishing between resistance/susceptibility status of the tested mosquito populations. Biochemical assay data (enzymatic activity per mg protein, levels of MFO, NSE, GST and AChE inhibition between Kisumu and Yaokoffikro *An. gambiae s.s.*) were compared using Mann-Whitney non-parametric *U*-test (Statistica software). Conformity of L1014F *kdr *and *ace-1 *G119S mutation frequency with Hardy-Weinberg expectations was tested for *An. gambiae s.s. *population from Yaokoffikro using the exact probability test [39]. Statistical significance was set at the 5% level.

## Results

### Bioassays

The susceptibility data on *An. gambiae s.s. *Kisumu and Yaokoffikro are gathered in Table 1.

**Table 1:** Bioassay mortality of *An. gambiae s.s. *population from Yaokoffikro and Kisumu strain

	Kisumu			Yaokoffikro		
	
Insecticides	N	% Mortality	Status	N	% Mortality	Status
0.4% Carbosulfan	99	100	**S**	79	13.9	**R**

0.1% Propoxur	100	100	**S**	113	22.1	**R**

1% Fenitrothion	102	100	**S**	97	94.8	**R**

1% Permethrin	101	100	**S**	106	68.9	**R**

0.05% Lambdacyalothrin	102	100	**S**	97	68	**R**

0.05% Etofenprox	105	100	**S**	100	36	**R**

4% DDT	98	100	**S**	99	12.1	**R**

N = number of tested mosquitoes

Status: resistance status were determined according to WHO [30]; S = susceptible,

R = resistant

Control mortality was consistently below 5%. All discriminating concentrations of the insecticides tested killed 100% of *An. gambiae s.s. *Kisumu, confirming susceptibility of this strain to the insecticides and the good quality of the impregnated papers.

Based on WHO criteria, the *An. gambiae s.s. *population from Yaokoffikro displayed resistance to all insecticides tested, (<69% mortality) except fenitrothion (95% mortality) (Table 1). However, resistance was more marked towards DDT and the two carbamates, with less than 23% mortality after 24 h holding period.

### Detection of resistance genes by PCR

Of the total number of mosquitoes (111) analysed for the L1014F *kdr *mutation, 108 (97%) were carriers of the mutation. The allelic frequency was high (0.94) for the L1014F *kdr *whereas it was 0.50 for the *ace-1*^R ^due to deficiency of homozygous.

### Biochemical assays

Table 2 shows the mean activity of MFO, NSE, GST and AChE inhibition rate of *An. gambiae s.s. *populations from Yaokoffikro *versus *susceptible reference Kisumu strain.

**Table 2:** Genotype frequencies of the *kdr*, *ace-1 *locus and mean level of NSE, MFO and GST activity in *An. gambiae s.s. *Kisumu and Yaokoffikro

	F(*kdr*)	F(*ace-1^R^*)	NSE μmol/mim/mg	MFO nmol P450 unit/mg	GST μmol GSH/mim/mg	AChE % inhibition
Kisumu			0.064 ± 0.022^a^	0.031 ± 0.015^a^	0.05 0 ± 0.035^a^	81.0 ± 12.4^a^
			(40)	(71)	(30)	(48)
Yaokoffikro	0.94	0.50	0.336 ± 0.190^b^	0.039 ± 0.037^b^	0.154 ± 0.115^b^	60.4 ± 19.1^b^
	(111)	(44)	(113)	(113)	(108)	(72)

Number of mosquitoes analysed in parentheses; Numbers in the same column sharing a letter superscript do not differ significantly (P > 0.05) at the 5% level

The mean NSE (0.336 ± 0.190) and GST (0.154 ± 0.115) activities were significantly higher in *An. gambiae s.s. *from Yaokoffikro than in the reference strain Kisumu (0.064 ± 0.022 for NSE and 0.050 ± 0.035 for GST) (P < 0.001). Such a 5-fold significant increase in NSE and 3-fold in GST in samples from Yaokoffikro suggest a strong involvement of these enzymes in insecticide resistance at this site. Although the samples from Yaokoffikro displayed mean MFO activity (0.039 ± 0.037) significantly higher than the level seen in susceptible Kisumu strain (0.031 ± 0.015, P = 0.007) (Table 2), the increase was only 1.25 fold. This was due to 2.7% outlier individuals displaying values over 0.09 mol EU mg^-1 ^protein.

The mean inhibition rate of the AChE in Yaokoffikro samples (0.60 ± 0.19) was significantly lower than in Kisumu (81 ± 12.4, P < 0.001), confirming the presence of an altered AChE responsible for carbamate and organophosphate resistance in this field population of Yaokoffikro.

## Discussion

Six years after the armed conflict in Côte d'Ivoire, this study was designed to update on resistance status of *An gambiae s.s. *at a previous WHOPES field site (Yaokoffikro), where a group of experimental huts have served for the evaluation of insecticides several years prior to the crisis. The results presented here show that insecticide resistance in this vector population is multifactorial and includes, in addition to target site mutations, enhanced metabolic mechanism component never identified before. At least four resistance mechanisms were found in the S form of *An. gambiae s.s. *at this locality: high frequency of the L1014F *kdr*, *ace-1^R ^*and increased activity of NSE and GSTs. The specific role of these enzymes in resistance has yet to be determined using more advanced techniques such as quantitative multiplex RT-PCR.

High resistance to DDT, pyrethroids and carbamates as detected previously was still present and the resistance profile did not change over the 6 year break [16,24,28,30]. This was because unaffected farmers did stick to their farming practices during the crisis. They continued to use pesticides to treat vegetables, which maintained the selection pressure, in conjunction with use of LLINs in the area. Previous studies in Benin, already demonstrated the influence of vegetable farming on selection of resistance in malaria vectors [6,40,41].

The present study identified a set of resistance mechanisms to pyrethroids and carbamates currently deployed for IRS and net treatments. Whether these mechanisms on their own and/or their combination thereof in *An. gambiae *s.s are associated with a fitness cost remains to be investigated. Since *kdr *and *ace-1^R ^*mutations in mosquitoes interact to positively or negatively influence a mosquito's fitness, both in the presence or absence of insecticides [42], additional interactions would suggest the dynamics of resistance will be difficult to predict in populations where multiple resistance mechanisms such as these are present or that are subject to treatment by different insecticides.

Pyrethroid resistance associated with cross-resistance to DDT is well documented in *An. gambiae s.s. *from Yaokoffikro, and is closely associated with L1014F *kdr *mutation [16,24,28]. The predominance of L1014F *kdr *was confirmed in the current study. It appeared to be highly conserved, with frequency (0.94) within the range of that reported previously (0.90), [24].

The results also confirmed carbamate resistance detected in *An. gambiae s.s. *populations from Yaokoffikro [29,30]. Resistance to the carbamates (carbosulfan and propoxur) in this vector was intensely present whilst only tolerance to the organophosphate (fenitrothion) was found. The mortality rates recorded were less than 22% with the carbamates and it was 95% with fenitrothion. The presence of cross-resistance to carbamates and to some minor extent to fenitrothion, suggests the presence of the *ace-1 *G119S mutation, further identified by PCR. Besides this phenotypic expression, the frequency of the *ace-1 *G119S in *An. gambiae *from Yaokoffikro was 0.50, similar to what was found by Asidi *et al. *[24]. However, although the *ace-1 *G119S mutation conferred cross-resistance to carbamates and organophosphates, the resistance level varied greatly between both insecticide families. Such variation could be due to the differences observed in the dominance level of the allele, as this mutation has been shown to be recessive with organophosphates but dominant towards carbamates [43].

Despite high L1014F *kdr *frequency found in *An. gambiae s.s. *(S molecular form) from Yaokoffikro, several trials of insecticide treated nets in that area repeatedly showed high mortality and continued protection against mosquito bites [18,21,22]. This contrasts with a type of pyrethroid resistance found in M molecular form of *An. gambiae s.s.*, (also associated with high L1014F *kdr*) in southern Benin that seems to be highly protective against pyrethroid effects [44,45]. The difference in pyrethroid toxicity between Benin and Côte d'Ivoire might lie in the resistance pattern found at both sites: metabolic resistance enhancing the resistance already caused by *kdr *include NSE and oxidases in south Benin [17,46] and GSTs, NSE in Côte d'Ivoire.

Glutathione S-transferases catalyse the dehydrochlorination of DDT to DDE and have been reported to be involved in DDT-resistance in many insects including *An. gambiae *[47]. The significantly higher level of DDT-resistance found in Yaokoffikro samples may be explained by the co-occurrence of GST and L1014F *kdr *at this site.

MFOs are involved in the detoxification of substrates and generally associated with resistance to pyrethroids [17,48,49]. The slightly increased level of MFOs seen in the present experiment would suggest their involvement in pyrethroid resistance at Yaokoffikro, although this seems to be a growing phenomenon at this site because only 2.7% of individuals showed activity higher than in the Kisumu strain. Further exploration establishing correlation between the activity of these enzymes, bioassay mortality and their inhibition with synergists would be necessary.

The involvement of L1014F *kdr*, *ace-1^R^*, NSE, GST and to a lesser extent MFO in resistance at Yaokoffikro stand in contrast with other field sites at Pitoa (Cameroon), where greater oxidase and esterase activities were observed in *An gambiae s.s. *[49,50] but *kdr *and *ace-1^R ^*were absent. So far, only L1014F *kdr *and *ace-1 *G119S mutations were observed in *An. gambiae s.s. *at Vallée du Kou in Burkina Faso [51]. These results suggest that each field site has its own characteristics regarding the diversity of vector populations and the resistance mechanisms they bear inside them. The degree of the threat that each of these complex mechanisms may pose to any new control intervention would vary according to geographical context.

## Conclusion

In addition to high L1014F *kdr and ace-1^R ^*previously detected and confirmed in the present experiment, highly significant increase in NSE and GST activities were found in *An. gambiae s.s. *from Yaokoffikro field site. Some increase in oxidase levels was also observed. The results suggest that a package of resistance mechanisms are present in this area of Côte d'Ivoire. Trials to evaluate their impact on the protective efficacy of malaria control interventions as well as new tools to manage these complex mechanisms are urgently needed. The site calls for innovative research on the behaviour of the local vector, its biology and genetics that drive resistance.

## Abbreviations

LLINs: Long Lasting Insecticidal Nets; IRS: Indoor Residual Spraying; WHOPES: World Health Organization Pesticide Evaluation Scheme; IPR: Institut Pierre Richet; L1014F *kdr*: west knockdown resistance; *ace-1^R^*: acetylcholinesterase-1 resistance; *ace-1 G119S*: G199S mutation in *ace-1^R^*; NSE: Non-specific esterase; MFO: Mixed-function oxidase; GST: Glutathione S-transferase; PCR: Polymerase chain reaction; DDT: Dichlorodiphenyltrichloroethane; DDE: Dichlorodiphenyldichloroethylene; R: Resistant; S: Susceptible; GSH: Reduced form of glutathione.

## Competing interests

The authors declare that they have no competing interests.

## Authors' contributions

AAK and LPAA, designed the study, conducted the field and laboratory work, the genotyping, interpreted the data and drafted the manuscript. MAA contributed to data analysis. MK and FC supervised and contributed to the manuscript draft. RN revised the study design and the manuscript critically for intellectual content. All authors read and approved the final manuscript.
